# Medication self-management in patients with epilepsy: a narrative review of current status, influencing factors, and intervention strategies

**DOI:** 10.3389/fneur.2025.1657256

**Published:** 2025-11-27

**Authors:** Huayuan Wang, Xinmin Liu

**Affiliations:** 1 School of Nursing, Jilin University, Changchun, China; 2 Department of Neurology, The First Hospital of Jilin University, Changchun, China

**Keywords:** epilepsy, antiepileptic drugs, self-management, influencing factors, review

## Abstract

The contribution of medication management to epilepsy treatment has been demonstrated in numerous studies. However, existing research has primarily focused on improving patient medication adherence and optimizing medication regimens by healthcare teams, lacking a systematic review of the current state of medication self-management from the patient perspective. This paper systematically reviews published research in the field of medication self-management among epilepsy patients, elucidating the concept of medication self-management, its current challenges, influencing factors, and advances in intervention strategies. The study concludes that a multidimensional intervention system integrating individual characteristics, technological empowerment, and systemic support must be established in the future. The efficacy of interventions should be validated through large-scale, long-term studies, while advancing the human-centered design of technological tools to achieve inclusive and precision-driven development in global epilepsy management.

## 1 Introduction

Epilepsy is a chronic brain disease state with different etiological bases and diverse clinical manifestations, but characterized by recurrent epileptic seizures, which are ictal, transient, stereotypical, and repetitive, severely affecting the quality of life of patients (1). Globally, the number of patients with epilepsy (PWE) is huge, affecting 50 million people of all ages worldwide (2). In China, there are about 9 million people with epilepsy, of whom about 6 million have active epilepsy, which is one of the most common neurological disorders (3).

**Figure 1:**
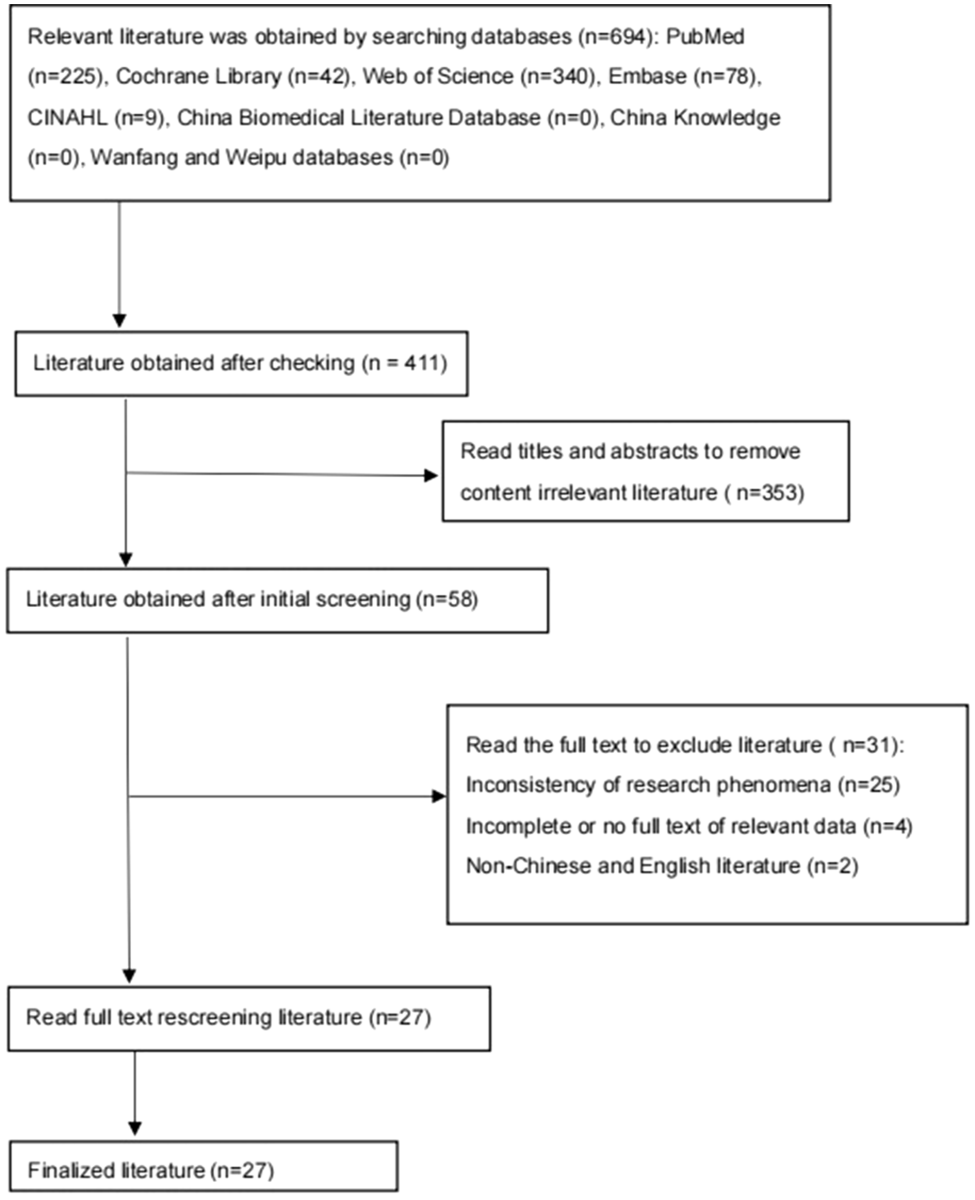
Literature screening and inclusion process.

The main form of treatment for epilepsy is antiepileptic drugs (antiepileptic drugs, AEDs), which result in seizure control in 60% to 70% of cases (4). Medication is the core tool for epilepsy management, and most patients require long-term or even lifelong medication. Long-term medication not only imposes a financial burden on patients but may also lead to recurrent disease due to medication side effects or irregular medication use. The ability of medication self-management directly determines the outcome of epilepsy control. In 25% of cases where the physician changes medication or adjusts the dosage, the real reason for poor seizure control is the patient’s adherence problem rather than the insufficiency of the medication itself (5). Nonadherence to medication typically reduces the quality of treatment outcomes, increases consultation and hospitalization rates, and raises healthcare costs (6). Despite the risks associated with medication non-adherence, 50% of patients with chronic conditions fail to comply with their treatment recommendations (7), which has been linked to the fact that AEDs have side effects such as drowsiness, irritability, and fatigue. As approximately half of epilepsy cases require combination medications, the issue of medication side effects may be further accentuated in such scenarios, requiring patients to implement effective medication self-management (see Figure 1).

Most existing systematic reviews have assessed self-management strategies at different ages (children, adolescents, adults) and for various types of epilepsy. Traditional adherence studies are overly reliant on patient self-reporting and often suffer from limitations such as a lack of attention to individual patient differences. In recent years, the research paradigm of medication self-management has been transformed with the intervention of novel technologies, such as mobile health apps and smart reminder devices, which provide new ideas to improve patient adherence and quality of life. Some studies have looked at advances in the use of internet technology in epilepsy diagnosis and management (8), such as the analysis of the association between seizure prediction and medication behavior by wearable devices. Some studies further investigated the effect of group self-management interventions on medication adherence in patients with epilepsy (9). The results were consistent, i.e., improving medication self-management in patients with epilepsy could bring benefits to patients, with significant improvements in health literacy, quality of life scales, and seizure frequency. Currently, most domestic and international studies focus on improving patients’ medication adherence and reducing patients’ medication burden by optimizing the medication regimen through the healthcare team and reducing healthcare costs, while epileptic patients are usually responsible for the daily medication management and are medication self-managers. It is of practical significance to construct a medication self-management program from the patient’s perspective, to adjust the medication plan according to their symptoms under the guidance of the doctor, and to negotiate with the doctor about the treatment plan according to the changes in their condition, to improve the ability of medication self-management and reduce the burden of medication use.

Therefore, this paper aims to reveal the evolution of trends in medication self-management research in patients with epilepsy by systematically combing through related studies, identifying evidence gaps in current research, and providing a reference for constructing an epilepsy medication self-management intervention system.

## 2 Concepts of medication self-management in patients with epilepsy

The concept of medication self-management stems from self-management, and medication self-management is a crucial aspect of chronic disease self-management (10). The concept of hospital medication self-management was first proposed by Parnell in 1959 and has been studied internationally for many years (11, 12). Although a considerable amount of research has been conducted on medication self-management among patients, there is still no universally accepted definition for the term “medication self-management.” Bailey (13) proposed a patient medication self-management model based on the medication adherence process and health literacy theory, which deconstructs medication self-management into a series of steps patients must undertake: obtain prescriptions and medications; understand daily medication use; schedule medication use around daily routines and plans; take medications correctly; monitor medication effectiveness and safety; maintain safe and appropriate medication use. According to this model, medication self-management is defined as the process by which patients take their prescribed medications, encompassing not only correct dosage, frequency, and intervals but also their continued safe use over time. The American Pharmacy Association (14) defines medication management as collaborative care between the patient and the healthcare team, utilizing the patient’s active role in managing his or her medications. It aims to optimize the benefits of medications and minimize potential harms by providing safe, effective, and appropriate medication regimens.

Ideal medication management includes medication review, health assessment, medication monitoring, medication regimen development, education, efficacy and safety of treatments, and promotion of patient self-management (15). Barlow et al (11) proposed that medication self-management is an individual’s ability to manage the symptoms, treatments, and physiological and psychosocial consequences inherent in chronic disease, as well as lifestyle changes. Tools and actions for medication management include high-quality medicines, medical devices, diagnostic tools, and digital interventions that can be partially or fully provided outside of formal healthcare and can be used with or without direct supervision by a health worker, covering a wide range of scenarios from mild symptom relief to chronic disease management (12).

WHO defines adherence as the consistency of behavior with medical advice, emphasizing implementation rather than decision-making. Compared to medication adherence, medication self-management is a broader, proactive concept that encompasses adherence and emphasizes the patient’s full participation and ability to make dynamic adjustments. Medication adherence, on the other hand, is a behavioral level of implementation that focuses on passive compliance with medical advice. The difference between the two is particularly significant in chronic disease management, which is a progression from passive execution to active management, emphasizing patient agency.

## 3 Current status and problems of medication self-management in patients with epilepsy

### 3.1 Current status

According to the World Health Organization (WHO), chronic diseases such as epilepsy pose the most significant challenge to modern health care systems (16). Nearly half of the hospitals in the United States have less than 100 available beds, a lack of accessible neurologists (17), inconvenient access to medical care for many patients with epilepsy in remote mountainous areas (18), inability to instruct patients with epilepsy in effective medication self-management, and lack of basic knowledge of epilepsy, such as etiology and treatments for most patients. A study by Wu et al. (19) in China found that the level of self-management of adult epilepsy patients varied, and most of them were able to grasp the general knowledge of the disease, such as epileptic triggers for seizures, but their ability of medication was relatively weak. A study of 126 epilepsy patients’ self-management ability in Saudi Arabia (20) found that more than half of the patients had insufficient knowledge about the use and safe management of their antiepileptic drugs. A study of 100 hospitalized epilepsy patients in Jinan, China (21) showed that 91% of the patients lacked knowledge about the proper handling of medications.

In summary, medication self-management in patients with epilepsy is generally weak globally, with significant geographic variation, exacerbated by inadequate access to healthcare resources. This suggests that nursing practice needs to focus on strengthening the medication management education capacity of primary care nurses, constructing a telecare follow-up system to compensate for geographic barriers, developing culturally appropriate medication management tools, and improving patients’ knowledge and self-management efficacy through nursing interventions, with a particular focus on precision interventions in resource-poor areas.

### 3.2 Problems

#### 3.2.1 Behavioral dimensions

Behavioral deficits in medication self-management among patients with epilepsy mainly include behaviors such as skipping doses, self-reducing medications, and repeating medications. A study of predominantly African American and Caribbean American patients with epilepsy showed (22) that 63% of patients had behaviors such as forgetting to take their medication and failing to take their medication on time, which was especially common with irregular daily medication times. In a Swedish cross-sectional study (23), 38% of patients missed at least one dose of antiepileptic medication per month, mainly due to forgetfulness, which accounted for 72% of non-adherence behaviors. In the African Ethiopian study (24), medication nonadherence was as high as 41.2%, where missed dose behavior was directly related to the perception of medication side effects. In addition, prolonged dosage intervals when medication was insufficient were common behaviors, especially in patients with untimely medication refills, accounting for 28% (22). Repeated medication is mostly seen in specific scenarios, for example, about 16.6% of patients with persistent status epilepticus repeat medication on their own to control seizure clusters (25), which is related to epileptic patients’ miscalculation of the severity of seizures and lack of medication knowledge (26).

#### 3.2.2 Cognitive level

Cognitive function indirectly affects patients’ behavioral decision-making and quality of life by mediating the relationship between symptom burden and adherence, which can effectively consolidate and improve medication adherence by decreasing symptom burden and improving cognitive functioning, and the lack of knowledge at the cognitive level is one of the core influences on the abnormalities of patients’ medication self-management ability. From a cognitive psychology perspective, patients’ lack of understanding of disease pathology and drug mechanisms affects the individual cognitive-behavioral chain, leading to decision-making bias and executive dysfunction. Motioleslam et al. (26) found that differences in perceptions of the necessity of medication significantly affected medication self-management behavior, and those with low self-efficacy were more likely to make unauthorized dosage adjustments, and their behaviors were correlated with excessive concern about the long-term harms of medication. Excessive concern. Crook et al. (22) showed that patients who perceived medications to be harmful or overused had a 3.2-fold increased risk of self-medication reduction, and those with a lack of knowledge of drug metabolism were more likely to make dose adjustment errors. Polish studies have shown (27) that 51% of patients believe that medication can be discontinued in the absence of seizures, and that there is a lack of awareness of the need for long-term medication in epilepsy.

#### 3.2.3 Psychological dimensions

Influenced by the characteristics of epilepsy disorders, patient and public misconceptions and understanding of epilepsy, and negative psychological emotions, epileptic patients are prone to a variety of physical and psychological comorbidities (28), and indirectly, medication self-management problems. Stigma is one of the most important factors hindering self-management. A survey from Europe showed that more than 56% of epilepsy patients reported that they had experienced stigma (29), and an Asian study showed that more than 20% of seizure-free patients continued to feel stigmatized even if they had been seizure-free for more than 2 years (30). Studies in China have reported (31) that stigma can lead to epilepsy patients leading to a decline in patients’ confidence in the cure of the disease, concealment of medication, and a decline in compliance behavior (32). High levels of disease shame can indirectly increase the risk of missed doses by decreasing the acceptance of medication support (33).

In summary, patients with epilepsy have many problems in medication self-management, which can be categorized into three levels: behavioral, cognitive, and psychological. These problems are intertwined and seriously affect patients’ medication self-management ability and treatment outcome, suggesting that nursing practice needs to correct cognitive bias through structured education, use intelligent reminder tools to reduce the rate of missed doses, introduce motivational interviewing to alleviate the sense of shame, and carry out cognitive interventions on seizure severity for patients with epilepsy status persistence, as well as strengthen the multidisciplinary team’s psychological support for patients with co-morbid depression.

## 4 Factors influencing medication self-management in patients with epilepsy

Medication self-management in patients with epilepsy is influenced by many factors, such as the patient’s attitudes and beliefs about medication and health, ability to pay for medication, and access to treatment. Medication adherence is an element of self-management, and methods of recognizing medication nonadherence and factors contributing to nonadherence have been extensively reviewed and will not be discussed further here. This section focuses on factors that influence a person’s cognitive and physical ability to safely and accurately manage their medications. The assessment of a person’s ability to manage his or her medication regimen should take into account factors related to the patient, the medication regimen, and the healthcare professional.

### 4.1 Patient related factors

#### 4.1.1 Gender

Gender differences significantly influence medication self-management. Men are more likely to balance occupational stress with medication side effects, whereas women rely on family support networks to enhance management efficacy. This difference in gender roles makes men more concerned with balancing work stress and medication side effects when self-managing their medications, and the dual tension between occupational role fulfillment and health management is a central constraint on their self-management behaviors. In women, they rely more on family-community support networks to optimize the efficacy of medication self-management through the structural optimization of the social support system. Meanwhile, Lee et al. (34) found that women were more affected by AED polytherapy than men, and that clinical interventions for medication self-management in epileptic patients need to consider gender specificity. A Korean study (35) reported that considering the impact of epilepsy on women’s marital fertility, women are more likely to experience psychological anxiety in epilepsy disease management and are more sensitive to the side effects of antiepileptic drugs, which in turn affects medication management behaviors.

#### 4.1.2 Age

Children with epilepsy whose medication self-management is highly dependent on their caregivers, with common problems including forgetting to take medication, failing to replenish medication as scheduled, and failing to take medication on time, etc., and whose current situation is characterized by a “parental knowledge gap - low management efficacy” (36), which is significantly correlated with parental education level and monthly family income. Low-income families are more likely to have difficulties in medication management (37). Meanwhile, caregivers’ mental health status (e.g., anxiety, depression) directly affects the quality of medication management in children (38).

Medication self-management in the adolescent stage is characterized by the paradox of high cognitive demand-low adherence, with common problems including failure to take medication on time (22) and insufficient knowledge of disease chronicity (39). As they grow up, adolescents have diminishing expectations of family self-management and a growing quest for independence and autonomy (40), as well as an increased propensity for risk-taking behaviors and sensitivity to peer influence (41). Adolescents with epilepsy are at higher risk for adherence compared to children and adults (42, 43).

Medication self-management in adults with epilepsy is a multidimensional game of social roles and disease management, moderated by multiple social factors such as occupational and family factors, with behavioral disorders predominating, and 57% of patients having problems such as forgetting to take their medication and failing to replenish their medication promptly (22).

Medication self-management in elderly patients with epilepsy faces a unique dilemma, i.e., the dual challenges of multiple medication burdens and the ability to manage them. Cognitive decline (MMSE <24) in elderly patients is an independent predictor of the ability to self-administer medication (44). Due to metabolic changes associated with aging, the elderly population is less tolerant of medication side effects, more susceptible to AED side effects, and physiologic decline leads to higher rates of medication errors and increased risk of drug interactions (45). The prevalence of epilepsy increases with age, from 0.7% at 55–64 years to 1.2% at 85–94 years, and often coexists with other health problems such as cerebrovascular disease or neurodegenerative disorders (46), requiring the administration of other medications that act on the central nervous system. The prevalence of co-morbidities is as high as 74.6% in China’s elderly population aged 60 years and older (47), which faces multiple medication burdens (48).

#### 4.1.3 Education level

Self-management level was significantly associated with education level, with those with higher education performing better in information management and lifestyle adjustment (49). Patients with higher levels of education tended to actively explore information about disease management and were less likely to have concurrent problems such as poor treatment adherence or mood swings, whereas those with lower levels of education were more likely to misinterpret medication instructions (50).

#### 4.1.4 Occupation

Compared to the unemployed, retired population and housewives, employed or schooled patients showed lower adherence in adhering to medication self-management, which was related to concerns about the disease taking up a significant amount of time from work and school, and side effects of the medication, such as drowsiness, interfering with normal life (51).

#### 4.1.5 Family support systems

Family support influences patients’ medication self-management behaviors directly or through synergistic effects with patients’ mental health.

Family functioning is positively correlated with the level of patient self-management (52). Family support directly influences medication self-management behaviors in patients with epilepsy by increasing patients’ confidence in medication management, improving adherence, and optimizing family resource allocation (53). Family members can significantly reduce the incidence of missed medications and inadequate medication reserves by assisting with medication reminders, medication reserve management, and emotional support (22, 38). Family members’ involvement in medication management can reduce the rate of missed doses by 34% (38). Meanwhile, high-quality family support can alleviate patients’ depression and anxiety symptoms (54), while improved psychological status further promotes patients’ active participation in medication management, and epilepsy patients without depressive symptoms are more inclined to adhere to their medication schedules (53).

In summary, patient-related factors such as gender, age, education level, occupation, and family support system significantly affect medication self-management in epilepsy patients. When analyzing the influencing factors of medication self-management in epilepsy patients and constructing an intervention system, a precise intervention framework based on patient-related factors should be considered. For male patients, the focus should be on occupational environment adaptation, and the balanced mechanism of occupational role-health management should be constructed through the integrated guidance of work stress management and side effect coping. For female patients, it is necessary to strengthen the professional empowerment of the family-community support network and enhance the self-management effectiveness through the structural optimization of the social support system. At the same time, it is necessary to consider the differences in the life cycle of epilepsy patients of different age groups. For pediatric patients, family medication education should be strengthened, and medication regimens should be simplified. For adolescent patients, a transitional care program should be implemented. For adult patients, cognitive-behavioral interventions for medication beliefs. For elderly patients, use multidisciplinary teamwork to optimize prescribing and strengthen social support. For low-education and in-school patients, strengthen the ability to improve medication self-management.

### 4.2 Factors related to medication regimen

Duration of illness significantly affects the level of medication self-management, and patients with ≥5 years of illness tend to show better medication self-management (55) which may be related to the gradual adaptation of patients to the demands of disease management.

However, the effect of seizure frequency on medication self-management is controversial. Tian et al. (55) showed that seizure frequency did not have a statistically significant effect on medication self-management, but other studies have pointed out (56, 57) that seizure control is one of the core goals of medication self-management, suggesting that poor seizure control may inversely reduce patient self-efficacy. This may be related to the different characteristics of the study samples with different measurement tools and bias between objective seizure recording and self-report. At the same time, there is bidirectional causality between seizure frequency and self-management. Frequent episodes may reduce self-efficacy and promote omission, while poor management exacerbates episodes, and the temporal relationship has been handled differently in different studies. In addition, mediators have significant moderating effects, and the inclusion or exclusion of variables such as self-efficacy and stigma in the model may influence the conclusions and lead to contradictory findings.

Co-morbidities indirectly affect medication self-management mainly by influencing mood, and psychiatric co-morbidities such as depression and anxiety increase the risk of missed doses. And refractory epilepsy due to neuroimaging abnormalities requires multiple medications, leading to significantly more difficult medication management (58). In addition, residual symptoms and co-morbidities are not conducive to medication self-management (59), and ineffective management of medication side effects increases the risk of self-medication reduction by 1.8-fold (24).

In conclusion, factors related to medication regimens, such as disease duration, seizure frequency, severity, and co-morbidities, significantly influence medication self-management in patients with epilepsy. A multidimensional approach is needed to build an epilepsy medication self-management intervention system that dynamically assesses the heterogeneity of disease duration, strengthens maintenance strategies for patients with long disease duration, and focuses on basic cognitive education for newly diagnosed patients. At the same time, precise interventions for co-morbid patients and stratified management of side effects are needed. When providing medication information to patients with epilepsy comorbid with cognitive impairment, the amount and format of the information is important, as well as the level of cognitive impairment of the patient and the level of education of his/her caregivers, the use of simplified polypharmacy regimens for elderly co-morbid cognitively impaired patients, the provision of tools to visualize drug interactions in patients with refractory epilepsy, and the provision of customized interventions for high-risk groups.

### 4.3 Healthcare professional related factors

Healthcare accessibility includes geographic barriers and medication discontinuation risk. Long distances to healthcare facilities and long waiting times reduce patient adherence (60), while about half of patients are at risk of medication discontinuation due to failure to plan for medication refills (61), and intervals between follow-up appointments of >3 months are significantly associated with untimely medication refills (26).

The quality of doctor-patient interaction is reflected in the trust relationship and educational interventions. Patient trust in physicians is positively associated with medication management behaviors (60, 62). Meanwhile, healthcare professionals can significantly improve patients’ medication self-management skills using methods such as psycho behavioral interventions, such as health education interventions based on the information-motivation-behavioral skills (IMB) model (54). The distribution of medical resources is mainly related to socioeconomic differences. Low-income patients are more prone to nonadherence behaviors due to medical cost pressure (24). Meanwhile, economically developed regions have more adequate medical resources and are more likely to make full use of mHealth tools to improve management behaviors (63).

Beyond physicians, dedicated healthcare professionals play a pivotal role in supporting epilepsy patients’ medication self-management, with epilepsy-specialized nurses and clinical pharmacists serving as key pillars of support. Evidence-based research confirms that ESNs play an irreplaceable role in patient medication self-management, home safety interventions, and the prevention of sudden unexpected death in epilepsy. In clinical practice, ESNs fulfill multiple roles, including those of consultants, educators, liaisons, researchers, and administrators. ESNs who undergo standardized training acquire solid professional knowledge and high-level clinical skills through systematic education, resulting in a 20% reduction in patient seizure frequency and a 30% decrease in unplanned hospitalizations (64). More importantly, ESN engagement also helps boost patients’ confidence in taking medication and improves their adherence, thereby enhancing their ability to self-manage their drugs (65). Clinical pharmacists primarily focus on optimizing the feasibility and safety of treatment plans to reduce the burden of self-management for patients (66). They can significantly enhance patients’ independent management capabilities by guiding side effect management, medication storage, and refill planning (67). Research indicates that pharmacists can resolve 62.7% of medication-related issues (68).

In summary, healthcare accessibility, the quality of doctor-patient interactions, the involvement of epilepsy nurse specialists and clinical pharmacists, and the allocation of medical resources collectively influence patients’ ability to self-manage their medication. When establishing a systematic medication self-management intervention framework in the future, the first step should be to leverage regional epilepsy management cloud platforms to integrate electronic prescriptions with remote pharmacy services. This approach will overcome geographical barriers and establish a digital medication discontinuation alert mechanism, providing the technological foundation for interventions. In building a specialized team, epilepsy-trained nurses should be integrated into routine follow-up care to provide ongoing support. Concurrently, clinical pharmacists should be actively involved in optimizing treatment plans, particularly in managing complex polypharmacy regimens, to ensure continuity of nursing care and the safety of treatment protocols. Second, medical-patient interactions and educational strategies should be optimized by improving communication mechanisms and implementing tiered, structured health education to enhance patients’ cognitive and behavioral capabilities. Finally, to achieve sustainable equity, efforts should focus on establishing regional resource coordination networks that provide targeted support to economically disadvantaged groups, thereby ultimately ensuring the rational allocation and precise coverage of healthcare resources.

## 5 Interventions for medication self-management in patients with epilepsy

### 5.1 Traditional interventions

#### 5.1.1 Educational interventions

Educational interventions are at the core of traditional interventions, including knowledge dissemination, skills training, and structured health education, which help patients establish scientific cognition by teaching the causes of epilepsy, the mechanism of action of medications, and the norms of medication administration.

Studies have shown that training on medication time management, medication refill schedules, and dosage adjustment principles can significantly improve medication adherence; however, knowledge education alone has limited improvement in medication beliefs and needs to be combined with behavioral interventions (22). Structured health education focuses on the development of a personalized medication plan and regular follow-up assessment and adjustment. A health management program based on the “plan-do-check-dispose” model can significantly improve patients’ self-management ability and medication adherence (69). Additionally, multi-component education led by epilepsy nurse specialists, combining written materials with face-to-face guidance, can enhance patients’ understanding of complex medication regimens (70). An RCT demonstrated that self-management education significantly reduced the frequency of self-medication (54). Xu et al. found (49, 57) that health education based on the information-motivation-behavior model promotes behavioral change by addressing key issues related to information, motivation, and behavioral skills, and increased on-time medication adherence by 31.7%, with the effect lasting up to 6 months after the intervention months.

#### 5.1.2 Behavioral interventions

Behavioral interventions play an optimizing role among traditional interventions. It includes correcting medication behavior, strengthening self-monitoring, and establishing feedback mechanisms.

For common behavioral disorders such as forgetting to take medication and delaying medication refills, interventions focus on establishing regular medication habits, such as through medication reminder tools (22). Epilepsy diaries are often used in traditional interventions to record medication use and seizure frequency, helping patients to identify medication effects and potential risks. Structured recording tools can enhance the systematic nature of monitoring and provide healthcare professionals with a basis for adjusting the regimen (70). Medication self-management training led by epilepsy nurses has improved medication effectiveness, adherence, and overall health outcomes among epilepsy patients (71).

#### 5.1.3 Psychosocial support

Psychosocial support plays a key role in traditional interventions, including intervening in medication beliefs and building family and community support networks.

Interventions based on psychotherapeutic theories, which can prompt patients to reduce medication avoidance due to disease stigmatization through management training, and to identify and overcome barriers that may impede medication self-management, are potentially effective ways to improve patients’ ability to self-manage their medications (6), whereas negative medication beliefs about the harmfulness of medications or overuse of medications by physicians are the main barriers to medication self-management in patients (22). A prospective, randomized multicenter pilot study examined (72) that motivational interviewing, as a psychotherapeutic approach, can enhance patients’ perceptions of the necessity of medications and reduce self-medication reduction behaviors due to concerns about side effects by helping patients to get rid of their uncertainty and enhance their self-efficacy (73). Accepting patients with high perceived necessity and low worry are less likely to engage in dose-adjustment behaviors during drug shortages (22). Research indicates that motivational interviewing led by epilepsy nurse specialists is particularly effective in addressing deliberate non-compliance by altering patients’ perceptions of medication (74).

Involvement of family members has been shown to enhance medication monitoring, especially for children, patients in the adolescent-to-adult transition, and patients with cognitive impairment (75). Following training in medication management skills under the guidance of clinical pharmacists, patients’ ability to self-manage their medications has significantly improved (38). Meanwhile, regular home visits by community health workers can provide ongoing support (76).

In summary, traditional interventions, with education, behavior modification, and psychological support as the three pillars, have formed a complete chain from knowledge transfer to behavioral monitoring. However, there is insufficient evidence for the effectiveness of educational programs in controlling seizure frequency (77), and the mechanism for maintaining long-term adherence has not been clarified, so continuous reinforcement strategies need to be explored. Several composite intervention frameworks focusing on educational interventions and integrating behavioral skills training and motivational cognitive modulation have been studied in patients with epilepsy, but these studies are usually small or lack a control group (78). In the future, individualized stratification strategies need to be further explored to strengthen the role of primary care, fill intervention gaps in under-resourced areas through low-cost, high-frequency support provided by primary health workers (76, 79), and explore individualized stratification strategies that integrate digital tools with traditional methods to improve the accessibility and sustainability of interventions. Clinical practice should identify patient needs and target medication beliefs to design intervention programs that go beyond a single knowledge infusion model.

### 5.2 Digitalization and technology-driven interventions

#### 5.2.1 mHealth technology

“mHealth apps” have multiple functions and great potential. Digital interventions can bridge cognitive deficits and simplify communication between healthcare services and patients horizontally through knowledge push and interactive learning, and vertically through real-time health assessment at the population and individual levels, which can help diagnosis and facilitate patient management, and make information sharing more efficient and convenient. There are more studies focusing on the use of mHealth for patient medication self-management, and there has been a large number of mHealth technologies applied to improve chronic care for patients with diabetes, hypertension, and other diseases (80).

The Epilepsy Medication Self-Management App can help PWEs to create and record medication lists, including the number of repetitions remaining, personalized dosages, how many days the supply will last, and alert reminders. Xie et al (81) in China found that after the use of home medication management APP for epilepsy patients, the mastery of epilepsy medication knowledge of patients in the test group was significantly higher than that of the control group, indicating that home medication management APP for epileptic patients can effectively enhance patients’ beliefs about medication, which is conducive to the mastery of anti-epileptic intermediary medication-related knowledge, and to improve the patients’ initiative to learn.

More epilepsy self-management mobile apps exist on the market today (82, 83). Apps such as Epilepsy Journal and My Seizure Diary have features such as passing photos of medication and recording side effects of medication, which provide epileptic patients with the opportunity to register the time of medication administration, the type of medication they receive, and the frequency of obtaining medication. Many mHealth tools have been applied to medication self-management for people with epilepsy with good results, but in general, they are still in a relatively early stage of development. Mohsen et al. (83) studied 22 apps related to epilepsy self-management in Android and iOS mHealth and found that the functionality of these apps concerning their medication management capabilities differed from each other. Most of the apps had features to support medication management, but had low installation rates. Pandher et al. (82) found that despite the growing number of epilepsy apps in the smartphone market, only a few of the apps offered tools such as seizure diaries and medication tracking. Meanwhile, digital tools are inadequately adapted for older and less literate groups, with only 12% of apps in the Vietnamese study including voice interaction (84).

#### 5.2.2 Telemedicine and monitoring

The core advantage of the telemedicine model is to crack the geospatial barriers through the digital technology architecture and promote the realization of cross-regional radiation coverage of high-quality medical resources. Studies have shown (8), that this model can effectively shorten the distance of medical service access for patients in remote areas, and through the construction of a telemedicine collaboration network, the specialty diagnosis and treatment capabilities of tertiary hospitals are extended to the primary medical terminals, thus reconfiguring the paradigm of medical resource allocation.

The telemedicine model in epilepsy is mainly used in real-time remote communication between hospitals and hospitals, including remote consultation, remote teaching and discussion, remote image diagnosis, remote electroencephalography diagnosis, remote examination, etc. It promotes the development of epilepsy medication management in the direction of precision and accessibility, and provides a new path of standardized diagnosis and treatment, especially for areas with scarce medical resources, which demonstrates dual values of optimizing the efficiency of medical resource allocation and enhancing the quality of patients’ survival. It has demonstrated dual value in optimizing the efficiency of medical resource allocation and improving the quality of patients’ survival. The model reduces patient transportation costs, improves follow-up rates, increases epilepsy treatment coverage by 37% in resource-poor areas of Africa (85), and achieves safety equivalence to traditional outpatient clinics in antiepileptic medication adjustments during pregnancy (86). Meanwhile, parents receive medication education through telepharmacy services, showing reduced levels of medication management anxiety (38).

Seizure frequency and medication duration correlation are monitored by wearable devices (e.g., smartwatches), which are combined with AI algorithms to generate personalized medication recommendations (87). Electroencephalogram (EEG) remote diagnostic system identifies subclinical seizures and guides medication dose adjustment (88). Electronic pillboxes (AdhereTech) combined with SMS reminders reduced missed dose rates by 42% (89).

In summary, current interventions show a trend from monoeducation to multimodality and personalization, and the application of digital and technology-driven interventions in epilepsy medication self-management has demonstrated significant potential, especially in terms of improving accessibility, optimizing medication adherence, and reducing healthcare costs. In the future, we need to focus on the user-friendly design of technological tools, innovation of interdisciplinary collaboration models, and policy support, and strengthen long-term efficacy and equity studies to achieve universal access and precision in epilepsy management globally. The “digital divide” effect of technological tools needs to be guarded against, and age-friendly digital intervention tools should be developed, while ethical considerations should be increased to avoid data leakage and other principal omissions. There is insufficient evidence that medication self-management in patients with epilepsy improves seizure frequency, and more long-term follow-up data are needed. It is recommended that a tiered progressive intervention be used in clinical practice to organically combine traditional educational tools with smart tools, as well as to enhance early assessment and intervention of medication beliefs. Future studies should focus on addressing the heterogeneity of intervention effects and long-term maintenance mechanisms.

## 6 Discussion

Although medication self-management in epilepsy patients has shown a trend of convergence between technology empowerment and precision management, focusing on digital intervention penetration, personalized medical practice, and standardization of interdisciplinary collaboration, current studies still have limitations, such as prominent heterogeneity of intervention effects and unknown long-term maintenance mechanisms. Considering the significant impact of individual patient differences, social support, and accessibility of healthcare resources, large-scale, long-period randomized controlled trials are still needed to verify the long-term effects and cost-effectiveness of different interventions in various types of epileptic patients, and whether the existing intervention protocols are fully applicable to different characteristic populations. In addition, there is a need to further explore the consistency between the efficiency of technological tools and patient acceptance in clinical practice, to study in depth the psychological mechanisms of medication self-management in patients with epilepsy, and to develop personalized intervention protocols that are applicable to different patient groups.

In summary, the global medication self-management capacity of epilepsy patients is generally weak and has significant geographical differences, and its influencing factors show multidimensional interactions. Interventions are shifting from the traditional model to a new paradigm of technological empowerment, precision, and cultural appropriateness, but there is still a need to break through the bottlenecks of behavioral adherence, knowledge gaps, and insufficient systemic support. In the future, it is necessary to focus on large-sample, long-cycle research, patient-centered care, integrate technology, education, and policy resources, and build a sustainable ecosystem for chronic disease management.
